# Relaxin and atrial natriuretic peptide pathways participate in the anti-fibrotic effect of a melon concentrate in spontaneously hypertensive rats

**DOI:** 10.3402/fnr.v60.30985

**Published:** 2016-04-12

**Authors:** Julie Carillon, Audrey Gauthier, Sandy Barial, Michel Tournier, Nathalie Gayrard, Anne-Dominique Lajoix, Bernard Jover

**Affiliations:** 1 EA7288 Université de Montpellier, Montpellier, France; 2 Bionov Research, Montpellier, France

**Keywords:** cardiomyocyte, endogenous antioxidant defense, fibrosis, oxidative stress, relaxin/insulin-like family peptide receptor 1

## Abstract

**Background:**

In spontaneously hypertensive rats (SHR), a model of human essential hypertension, oxidative stress is involved in the development of cardiac hypertrophy and fibrosis associated with hypertension. Dietary supplementation with agents exhibiting antioxidant properties could have a beneficial effect in remodeling of the heart. We previously demonstrated a potent anti-hypertrophic effect of a specific melon (*Cucumis melo* L.) concentrate with antioxidant properties in spontaneously hypertensive rats. Relaxin and atrial natriuretic peptide (ANP) were reported to reduce collagen deposition and fibrosis progression in various experimental models.

**Objective:**

The aim of the present investigation was to test the hypothesis that, beside reduction in oxidative stress, the melon concentrate may act through relaxin, its receptor (relaxin/insulin-like family peptide receptor 1, RXFP1), and ANP in SHR.

**Design and results:**

The melon concentrate, given orally during 4 days, reduced cardiomyocyte size (by 25%) and totally reversed cardiac collagen content (Sirius red staining) in SHR but not in their normotensive controls. Treatment with the melon concentrate lowered cardiac nitrotyrosine-stained area (by 45%) and increased by 17–19% the cardiac expression (Western blot) of superoxide dismutase (SOD) and glutathione peroxidase. In addition, plasma relaxin concentration was normalized while cardiac relaxin (Western blot) was lowered in treated SHR. Cardiac relaxin receptor level determined by immunohistochemical analysis increased only in treated SHR. Similarly, the melon concentrate reversed the reduction of plasma ANP concentration and lowered its cardiac expression.

**Conclusions:**

The present results demonstrate that reversal of cardiac fibrosis by the melon concentrate involves antioxidant defenses, as well as relaxin and ANP pathways restoration. It is suggested that dietary SOD supplementation could be a useful additional strategy against cardiac hypertrophy and fibrosis.

Left ventricular hypertrophy is an adaptive response to pressure or volume overload that preserves cardiac function. Cardiac hypertrophy may also be considered as a strong predictor of cardiovascular events in clinical hypertension (1). In spontaneously hypertensive rats (SHR), a widely studied model of human essential hypertension, oxidative stress appears to be involved in the development and maintenance of hypertension and its associated cardiac remodeling (2, 3). Dietary antioxidant supplementation could therefore be a potential therapy. Indeed, some authors have demonstrated beneficial effects of agents with antioxidant properties in cardiovascular disorders (4–7). Beside treatments with dietary antioxidants such as selenium or vitamins, an original way to decrease oxidative stress is by inducing endogenous antioxidant defense. In this context, a potent anti-hypertrophic effect of a specific melon concentrate, able to increase endogenous antioxidant enzymes, has been previously demonstrated in the heart of SHR (8). However, the precise mechanism of action of this melon concentrate in the reduction of cardiac hypertrophy remains to be explored.

Together with hypertrophy, fibrosis of the cardiac tissue is also a common alteration observed in SHR rats (9, 10). Fibrosis serves as a compensatory response to myocardial injury and is defined as the hardening or scarring of tissues attributable to an excessive deposition of extracellular matrix components. It results from chronic inflammation and aberrant wound healing, and it is the final common pathway to almost all forms of progressive cardiovascular disease (11, 12). Interstitial fibrosis in hypertrophied hearts leads to cardiac stiffness and may alter the diastolic function as demonstrated in the SHR in which a role of fibrosis in the transition to failure was suggested (9). Moreover, the SHR presents an increased incidence of atrial arrhythmias attributed to myocardial fibrosis (13).

Among the anti-fibrotic peptides, relaxin was reported to reduce collagen type I and III expression in cardiac fibroblasts (14). Heart is both a source and a target organ for the relaxin hormone (15, 16). The activation of relaxin/insulin-like family peptide receptor 1 (RXFP1) by relaxin binding plays a role in extracellular matrix remodeling (16). Overall, relaxin has a high anti-fibrotic effect on preventing and reducing aberrant collagen deposition and fibrosis progression in various experimental models, regardless of pathogenesis (10, 16, 17). Another peptide involved in cardiac remodeling that may have a beneficial influence is the atrial natriuretic peptide (ANP). Indeed, some authors have shown that ANP could play a role in preventing cardiac disorders, such as fibrosis (18, 19).

Therefore, the present investigation was designed to further characterize the influence of a chronic supplementation with a specific melon concentrate on cardiac fibrosis in the SHR and its normotensive counterpart, the Wistar Kyoto rat (WKY). A specific objective was to assess the involvement of relaxin, RXFP1, and ANP as targets of melon concentrate that can participate in its beneficial influence on cardiac remodeling in genetic hypertension.

## Materials and methods

### Preparation and characterization of the melon concentrate

SODB (Superoxide dismutase by Bionov, Bionov, Avignon, France) is a dried melon juice concentrate, particularly rich in superoxide dismutase (SOD), resulting from a patented process.

Around 625 kg of a specific proprietary and non-genetically modified organism (GMO) melon variety *Cucumis melo* L. (equivalent to 15 kg of dried melon pulp) is needed to produce 1 kg of this dried melon juice concentrate. Briefly, the melon pulp is separated from skin and seeds and crushed before centrifugation. Then, the melon juice undergoes filtration and concentration steps. Finally, the obtained melon juice concentrate is freeze-dried. For nutraceutical applications, this freeze-dried melon juice concentrate is coated with palm oil, using the spray drying method, in order to preserve SOD activity from the digestive enzymes secreted above the small intestine.

Detailed information about the antioxidant content of this particular melon concentrate has been published in a previous study (20).

### Experimental design

This study was carried out in strict accordance with the Helsinki Declaration of 1975, as revised in 2008 and complied with the European and French laws (permit numbers B-3417226 and 34179). The protocol was approved by the French National Committee on the Ethics of Animal Experiments (n° 1109). All surgery was performed under ketamine and xylazine anesthesia, and all efforts were made to minimize suffering.

Twenty 9-week-old SHR and twenty 9-week-old WKY (Janvier, le Genest-St-Isle, France) were used in the present experiments. They were housed at 22±1°C, subjected to a 12 h light/12 h dark cycle with free access to both food (A04, SAFE, Augy, France) and tap water. Rats were assigned to four treatment groups (*n*=10 in each). One group of SHR and one group of WKY received the melon concentrate at the daily dose of 4U SOD mixed with food. The two other groups of SHR and WKY remained untreated and served as controls. Melon concentrate treatment was given for 4 days. The dose and duration of melon concentrate treatment were chosen according to previous studies where these conditions were efficient on cardiac mass, that is, the smallest dose within the shortest period of administration with a significant effect on cardiac mass (8). At the end of the experimental period, rats were weighed and anesthetized (ketamine and xylazine, 75 and 25 mg/kg). Then, 5 mL of blood was sampled in all rats by cardiac puncture, centrifuged (4,000 rpm, 10 min), and plasma was stored at −80°C until analysis. The left ventricle (LV) was weighed, and LV weight index (LVWI) was calculated (LVW to body weight ratio). The LV was cut into several pieces for various determinations.

### Histology

One piece of LV was paraffin-embedded and cut into 3 µm sections and mounted on Superfrost-Plus glass slides (Menzel, Braunschweig, Germany). For fibrosis determination, LV sections were stained with 0.1% picrosirius red and mounted in Eukitt medium. Fibrosis was quantified in five to ten given fields per animal, and expressed as the percentage of fibrous tissue area stained with picrosirius red. To determine the cardiomyocyte size, the shortest transverse diameter was measured in a blind fashion by three different observers on at least 10 transverse sections per heart.

For immunohistochemistry, LV sections were deparaffinized and immersed in citrate buffer (pH 6.0) for 20 min at 90°C. Endogenous peroxidase was blocked using freshly prepared 0.1% peroxide in water for 30 min, and then unspecific binding sites were blocked by blocking solution (10% normal goat serum, 1% bovine serum albumin in phosphate-buffered saline) for 20 min at 37°C. The slides were incubated at 4°C overnight with the anti-RXFP1 antibody (Sigma-Aldrich, Saint-Quentin Fallavier, France), 1:50 dilution, or with the anti-nitrotyrosine antibody (Merkmillipore, Fontenay sous Bois, France), 1:100 dilution. Detection was carried out with the novostain universal detection kit (Novocastra Laboratories, Newcastle upon Tyne, UK) and diaminobenzidine as chromogen. Hematoxylin was used as counterstain. RXFP1 and nitrotyrosine were quantified in five to ten given fields per animal. All analyses were performed using image analysis software (ImageJ).

### Cardiac Western blot analysis

The LV protein extraction was carried out on ice in 20 mM of Tris buffer (pH 6.8) containing 150 mM sodium chloride, 1 mM EDTA, 1% Triton 20%, 0.1% SDS, 1% protease inhibitor cocktail (Sigma-Aldrich). After centrifugation (1,500 rpm, 15 min at 4°C), the supernatant was collected and the extracted tissue proteins were then separated by SDS polyacrylamide gel electrophoresis. Equal amounts of proteins were loaded onto a 15% acrylamide gel with a 4% stacking acrylamide gel. Migration was conducted in a Tris-glycine-SDS buffer. After separation, proteins were transferred onto nitrocellulose membranes (Whatman, Germany).

Antioxidant proteins were detected by Western blot analysis. The primary antibodies against rat Cu/Zn-SOD, Mn-SOD, glutathione peroxidase (GPx), catalase (CAT), RXFP1, ANP, relaxin, and the control protein glyceraldehyde 3-phosphate dehydrogenase (GAPDH) were purchased at R&D Systems (Lille, France) or Sigma-Aldrich. Expression of GAPDH was used for checking the equal protein load across gel tracks. Secondary antibodies (Sigma-Aldrich), coupled with alkaline phosphatase, were used for revealing the primary antibodies. Band densities were obtained by scanning the membranes. Image analysis (ImageJ) was used for quantification after standardization within membranes by expressing the density of each band of interest relative to that of GAPDH in the same lane. Results are then expressed as percent of values obtained in untreated animals.

### Plasma immunoassay measurements

Plasma ANP and relaxin levels were assessed using enzyme immunoassay kits from Sigma-Aldrich and R&D Systems.

### Statistical analyses

Values are presented as means±SEM. Statistical analysis of the data was carried out using R software (R Foundation for Statistical Computing, Vienna, Austria) by one-way analysis of variance followed by Mann–Whitney's protected least significant difference tests. P-values less than 0.05 were considered to be significant.

## Results

### Melon concentrate supplementation reduced SHR-induced cardiac alterations

As shown in Table 1, no difference was observed in body weight whatever the group. SHR displayed an increase in LVW, LVWI, and cardiomyocyte diameter (by ~25%), compared with WKY rats. Melon concentrate supplementation reduced these cardiac alterations in SHR (Table 1). Indeed, SHR-associated higher LVWI was significantly decreased in SHR-treated group. Of note, cardiomyocyte diameter in SHR treated by melon concentrate did not differ from both WKY groups.

**Table 1:** Cardiac hypertrophy and oxidative status in SHR and WKY after melon concentrate administration

	WKY		SHR	
		
	Untreated	Treated	Untreated	Treated
BW (g)	277±5	271±3	272±2	278±3
LVW (mg)	569±11	564±8	709±8*	657±8*§§
LVWI (mg HW/gBW)	2.06±0.02	2.08±0.02	2.61±0.03*	2.36±0.02*§
Cardiomyocyte diameter (µm)	17.8±0.6	17.6±0.7	22.4±0.6*	18.0±0.6§
Nitrotyrosine (% of stained area)	1.0±0.1	1.1±0.1	3.7±0.5**	2.1±0.3*§
SOD (% of untreated group)	100	113±3§	83±2*	99±2§
GPx (% of untreated group)	100	114±4§	86±3*	101±2§
CAT (% of untreated group)	100	119±6	102±3	96±7

Values are means±SEM. BW, body weight; LVW, left ventricle weight; LVWI, left ventricular weight index; SOD, superoxide dismutase; GPx, glutathione peroxidase; CAT, catalase.

SOD, GPx, and CAT protein expressions were standardized within membranes by expressing the density of each band of interest relative to that of GAPDH in the same lane. Results are then expressed as relative change from the corresponding untreated group (WKY or SHR) band intensity.

* *p*<0.05

** *p*<0.01 compared with untreated WKY

§ *p*<0.05

§§ *p*<0.01 effect of melon concentrate treatment, compared with the untreated group.

**Fig. 1:**
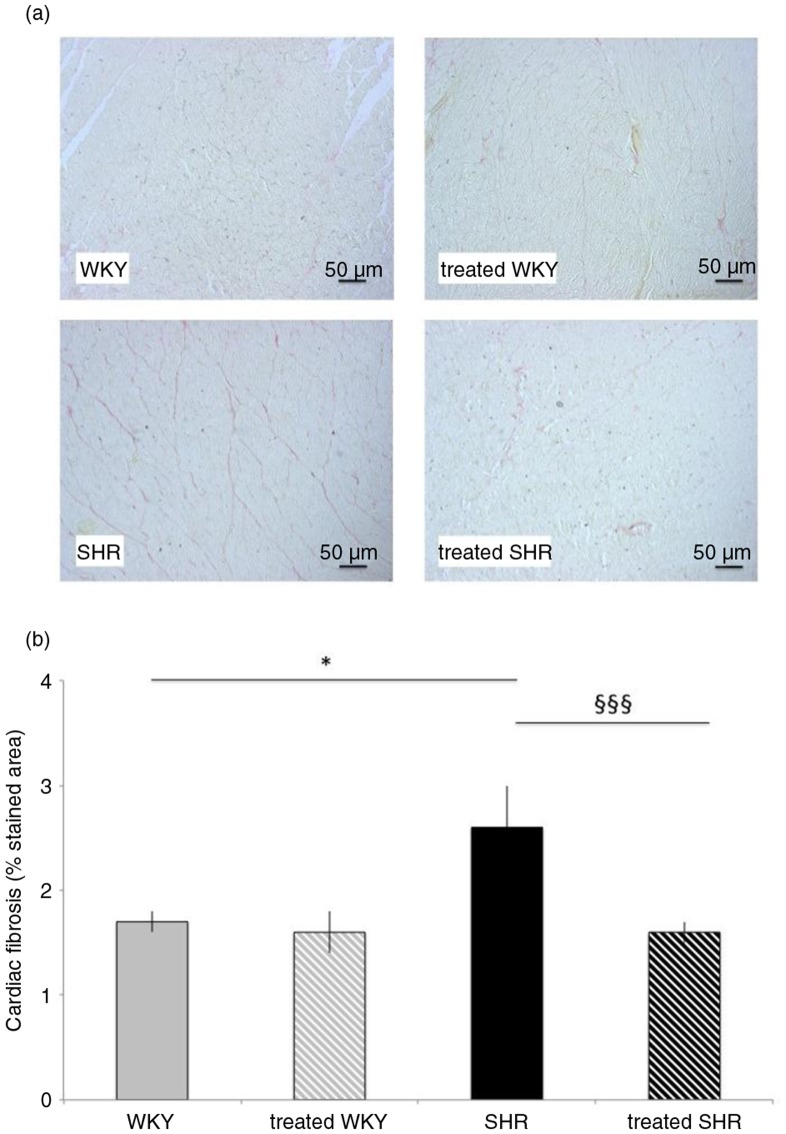
Influence of melon concentrate supplementation on cardiac fibrosis. (a) Cardiomyocyte staining with Sirius red. (b) Collagen content determined after staining with Sirius red on at least seven sections per heart. *p<0.05 compared with untreated WKY; ^§§§^*p*<0.001 effect of melon concentrate treatment, compared with the untreated group.

As presented in Fig. 1, staining of cardiac collagens was also higher (by 53%) in SHR, compared with the WKY group. SHR-associated collagen increase was fully corrected by melon concentrate supplementation (Fig. 1).

Melon concentrate treatment did not modify WKY cardiac characteristics, that is, LVW, LVWI, cardiomyocyte diameter, and collagen-stained area (Table 1).

### Melon concentrate supplementation modulated oxidative status in SHR and WKY

As shown in Table 1, nitrotyrosine-stained area was more than threefold larger in the heart of SHR, compared with the WKY group. Melon concentrate supplementation significantly decreased this oxidative stress marker in SHR (by 45%), compared with untreated SHR. No difference in nitrotyrosine-stained area was observed between WKY and WKY-treated groups (Table 1).

Cardiac antioxidant enzymes measurements by Western blot displayed a decrease in SOD and GPx protein expression in SHR compared with the WKY group (by 17 and 14%, respectively). These enzymes were induced after melon concentrate administration whatever the strain. Indeed, endogenous SOD and GPx protein expression was increased, by 13 and 14% in WKY rats, and by 19 and 17% in SHR animals, respectively (Table 1). CAT protein expression was not modulated whatever the group.

### Melon concentrate supplementation corrected SHR-altered relaxin pathway

**Fig. 2:**
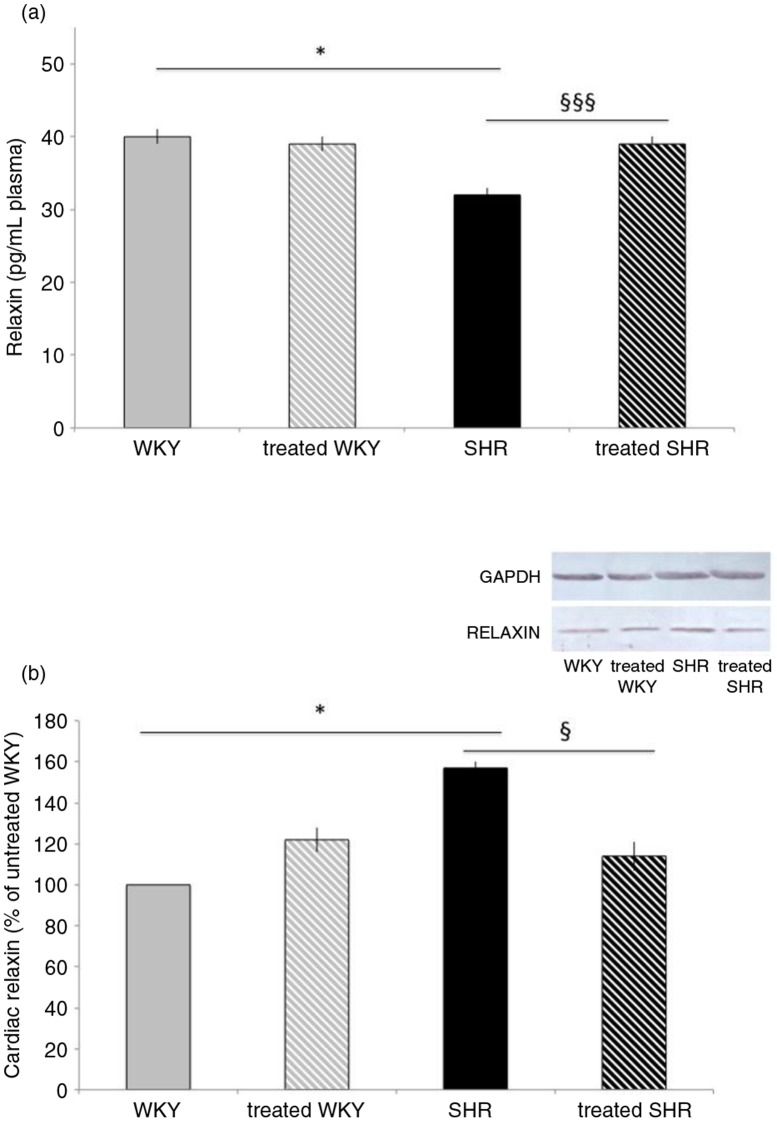
Influence of melon concentrate supplementation on relaxin concentration. (a) Plasma relaxin level. (b) Cardiac protein expression of relaxin. Quantification was made after standardization within membranes by expressing the density of the band of relaxin relative to that of GAPDH in the same lane. Results are then expressed as relative change from untreated WKY band intensity. *p<0.05 compared with untreated WKY; ^§^*p*<0.05 and ^§§§^*p*<0.001 effect of melon concentrate treatment, compared with the untreated group.

As shown in Fig. 2a, plasma relaxin concentration was decreased by 20% in SHR animals, compared with WKY. Melon concentrate treatment corrected this alteration by increasing circulating relaxin in SHR-treated group. No difference was observed in plasma relaxin concentration in the two WKY groups.

As depicted in Fig. 2b, cardiac relaxin measured by Western blot analysis was increased in SHR (by 57%), compared with WKY. Melon concentrate supplementation fully corrected the level of cardiac relaxin in SHR, whereas no difference was observed in WKY groups.

### Melon concentrate supplementation corrected SHR-altered RXFP1 concentration

**Fig. 3:**
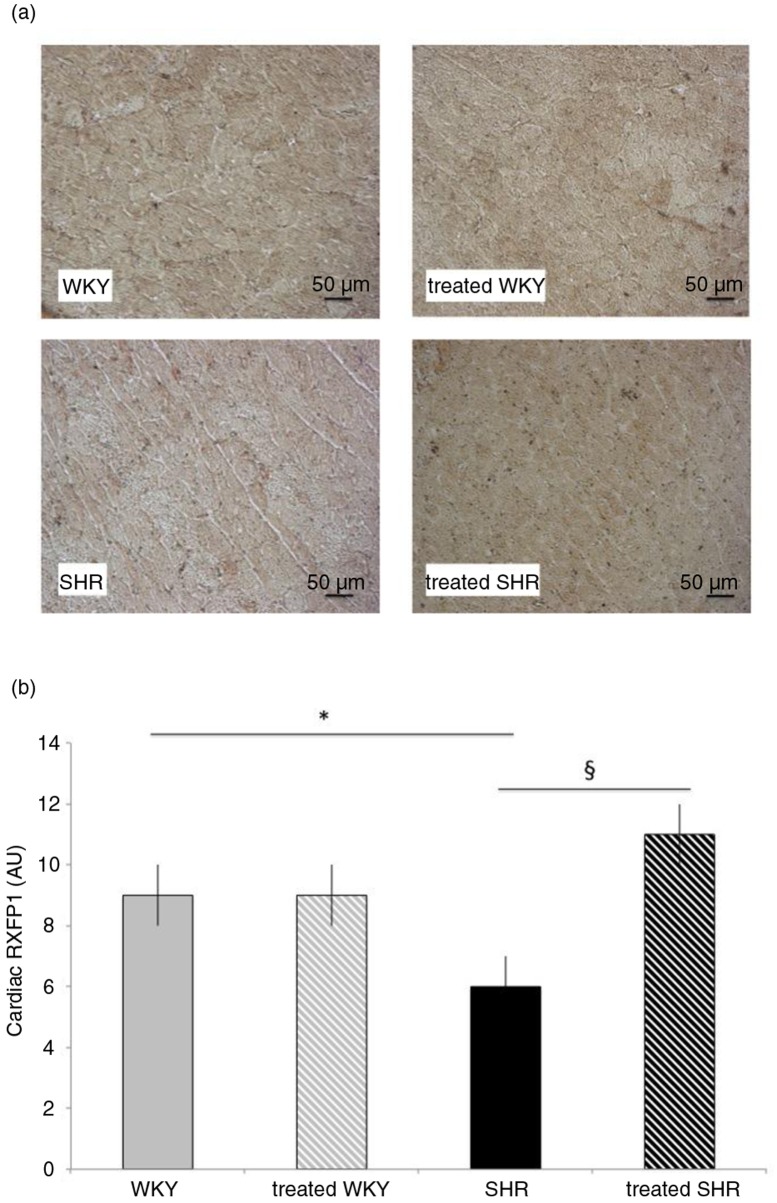
Influence of melon concentrate supplementation on RXFP1 concentration. (a) Immunohistochemical staining of cardiomyocytes with RXFP1. (b) RXFP1 level determined after immunohistochemical analysis on at least seven transverse sections per heart. *p<0.05 compared with untreated WKY; ^§^*p*<0.05 effect of melon concentrate treatment, compared with the untreated group.

Figure 3 represents the cardiac level of the relaxin receptor, RXFP1, which was significantly impaired in the heart of SHR, that is, 33% lower when compared with the WKY rats. Melon concentrate treatment fully corrected the level of this receptor in SHR, whereas no difference was observed in WKY groups.

Western blot results confirm the effect of melon concentrate administration. Indeed, cardiac RXFP1 level was significantly higher in SHR-treated group (111±10%), compared with the untreated SHR (64±9%).

Finally, no difference was observed in cardiac RXFP1 level between WKY (fixed to 100%) and WKY-treated groups (109±12%).

### Melon concentrate supplementation corrected SHR-altered ANP pathway

**Fig. 4:**
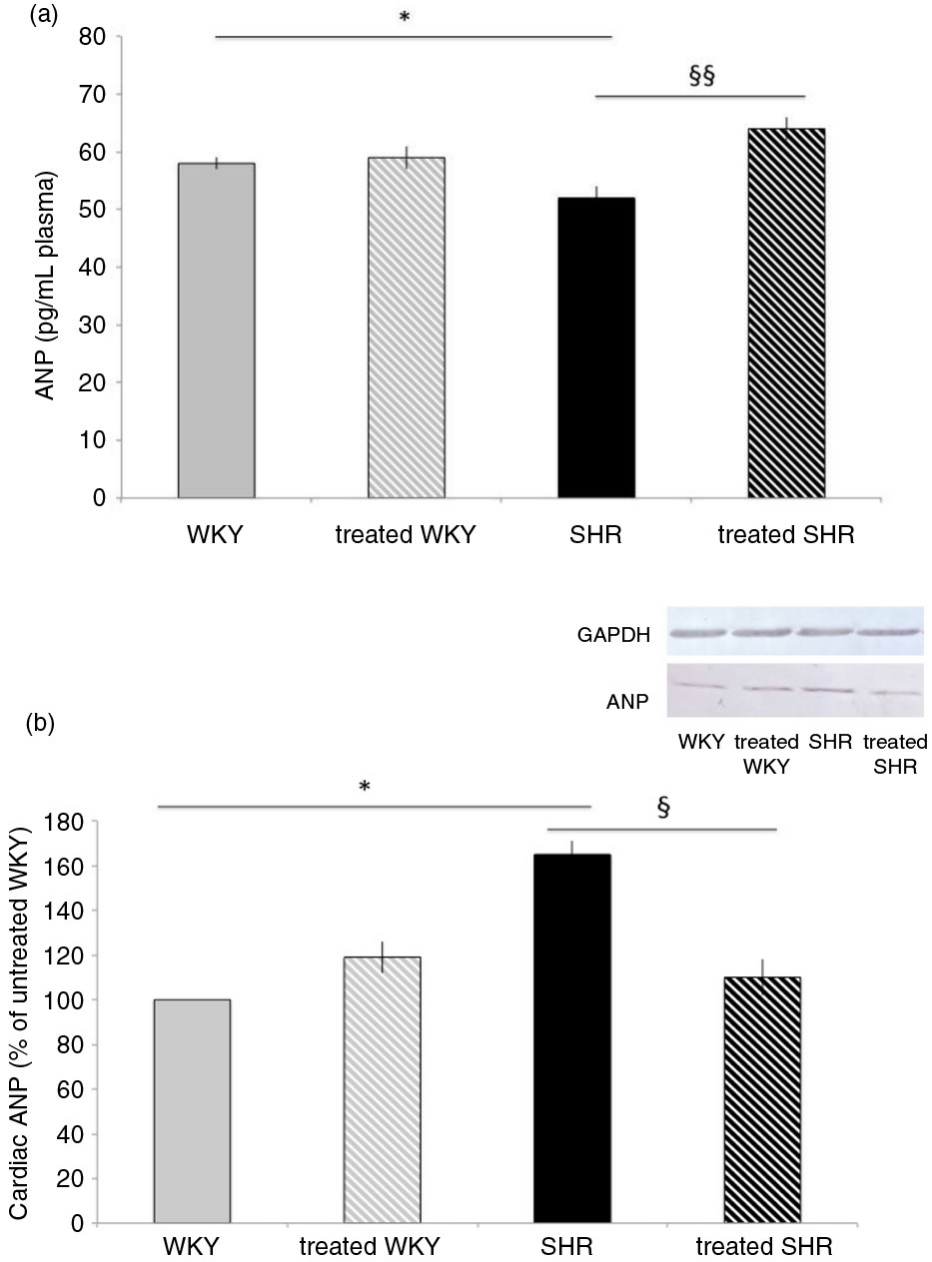
Influence of melon concentrate supplementation on ANP concentration. (a) Plasma ANP level. (b) Cardiac protein expression of ANP. Quantification was made after standardization within membranes by expressing the density of the band of ANP relative to that of GAPDH in the same lane. Results are then expressed as relative change from untreated WKY band intensity. *p<0.05 compared with untreated WKY; ^§^*p*<0.05 and ^§§^*p*<0.01 effect of melon concentrate treatment, compared with the untreated group.

Plasma ANP measurement (Fig. 4a) displayed the same variations as those of plasma relaxin, that is, a reduction in plasma concentration in SHR (by 10%), compared with WKY, and a correction with melon concentrate supplementation in SHR-treated group. No difference was observed in plasma ANP concentration in the two WKY groups.

As depicted in Fig. 4b, cardiac ANP measured by Western blot analysis was increased in SHR (by 65%), compared with WKY. Melon concentrate supplementation fully corrected the level of cardiac ANP in SHR, whereas no difference was observed in WKY groups.

## Discussion

The present results confirm the previous report (8) demonstrating in SHR that the specific melon concentrate could reverse cardiac hypertrophy, equated with LVWI and histological determination of cardiomyocyte diameter. Although arterial pressure was not recorded in the present experiment, we previously observed a weak anti-hypertensive effect of melon concentrate, which has probably only a minor influence on the reduction of cardiac mass in SHR (8). Cardiac hypertrophy regressed rapidly and to the extent that was observed in the previous report. In addition, this was the peak effect since no additional reduction was detected after 28 days of treatment with melon concentrate (8). Such a rapid regression of cardiac mass is not unusual and was reported in SHR after 1 week of AT1 receptor antagonist treatment (21) as well as within 1–3 days after aortic constriction relief in rats (22). Beyond pharmacological or mechanical blood pressure reduction, it was suggested that regression of cardiac hypertrophy is not merely a reversal but also a separate process with specific pathways; whether this occurred with the melon concentrate remains to be investigated. As expected, cardiac remodeling in the SHR was associated with enhanced fibrosis, evidenced by an increase in cardiac collagen staining. While melon concentrate treatment did not modify cardiac structure in WKY rats, it fully corrected fibrosis in SHR. Indeed, after 4 days of supplementation with melon concentrate, cardiac collagen staining by Sirius red was similar between WKY groups and the SHR-treated group. The current observations extend the beneficial effect of this particular melon concentrate to cardiac fibrosis, another deleterious alteration of the heart often associated with hypertensive states.

Among the various participants, which could be involved in the reduction of cardiac fibrosis, is the lowering of oxidative stress. Oxidative stress is increased in hypertension, cardiac fibrosis, and hypertrophy (23–25). An increase in reactive oxygen species (ROS) plays a critical role in the development and progression of cardiac remodeling (24, 25). As expected, cardiac oxidative stress was increased in SHR compared with WKY as evidenced by the higher nitrotyrosine level and the lower antioxidant defense expression. Interestingly, melon concentrate administration was associated with an increased expression of endogenous SOD and GPx in both SHR and WKY. This induction of antioxidant defense in the heart of SHR could naturally allow a decrease in oxidative stress, as previously shown in several studies in heart (8), liver (26), and adipose tissue (27). Here, we demonstrated a reduction of nitrotyrosine in SHR-treated group, which confirms and extends our previous report (8). The decrease in oxidative stress after melon concentrate supplementation could consequently explain a reduction of cardiac fibrosis in SHR.

While melon concentrate treatment did not modify cardiac remodeling in WKY rats, it induced endogenous antioxidant defenses in these control animals, as in SHR rats. It seems therefore that the increase of defenses is inactive without oxidative stress (as suggested in our study with low nitrotyrosine level in WKY) but could have a role during later oxidative damage. These results suggest that melon concentrate supplementation could induce antioxidant defenses independently of tissues or pathologies.

Another mechanism that may have an important role in the cardiac fibrosis is the modulation of relaxin pathway in SHR-associated cardiac disorders. In the present experiments, we observed an increase in cardiac relaxin and a decrease in circulating relaxin in SHR compared with WKY. The lower concentration of RXFP1 in SHR confirmed the relaxin pathway alteration in the hypertensive animals. The decrease in cardiac RXFP1 and modifications in relaxin in SHR could therefore facilitate cardiac fibrosis. Relaxin administration was shown to reduce cardiac fibrosis in the hypertrophied heart associated with β-adrenergic system enhancement (28) as well as in a model of diabetic cardiomyopathy (29). However, the role of endogenous relaxin is not unequivocal. In a model of hypertrophy induced by transverse aortic constriction, myocardial fibrosis was similar in relaxin-deficient and wild-type mice, thus suggesting that endogenous relaxin does not have a pivotal role in a chronic pressure overload model (30). In the absence of change in mRNA levels of RXFP1, it was suggested in the latter study that the increase in relaxin expression did not reach a level sufficient enough to offset the extensive fibrosis in this model. Yet, in the SHR model, endogenous relaxin was upregulated in cardiac tissue (31).

In the present SHR, the reduction of cardiac fibrosis by the melon concentrate was accompanied by an increase in plasma relaxin, to a level similar to that of normotensive rats. Various sources of relaxin were identified in the male rats, including the heart (32). In SHR, gene expression of relaxin and protein content of the peptide was elevated only in the heart (31). It is therefore plausible that the hypertrophied heart of the SHR was an important source of relaxin. Interestingly, cardiac relaxin was decreased after melon concentrate treatment in SHR while this treatment was devoid of influence in WKY rats. These observations favor an induction by the melon concentrate of relaxin secretion from the cardiomyocyte of SHR. Concomitantly, the cardiac content of RXFP-1, which was reduced in SHR, markedly increased after melon concentrate administration. Altogether, these results suggest that the beneficial effect of the melon concentrate in the SHR could be linked to the normalization of relaxin secretion and upregulation of its receptor, RXFP-1, that very likely improve the anti-fibrotic effect of the peptide.

In our study, cardiac ANP was increased whereas circulating ANP was decreased in SHR compared with WKY. These observations suggest that, as for relaxin, ANP pathway was altered in SHR. The dysregulation could also participate in the increase of cardiac disorders in SHR. ANP is often upregulated in cardiac ventricle alterations such as fibrosis (33–36). In studies using cultured neonatal myocytes and fibroblasts, exogenous administration of both ANP and ANP antagonists demonstrated that ANP has anti-fibrotic functions (37). Moreover, chronic ANP treatment decreased fibrosis in the heart of the SHR (18). Interestingly, here, melon concentrate supplementation was devoid of effect in WKY but corrected alterations in SHR, as observed for relaxin. Indeed, circulating ANP was increased whereas cardiac ANP was decreased in SHR treated with the melon concentrate.

Many studies in animal models have revealed a convoluted network of signaling cascades and transcriptional factors in cardiac fibrosis. The current observations suggest that the correction of relaxin and ANP secretion has a non-negligible role in the anti-fibrotic influence of the melon concentrate. In addition, the present results not only explain the decrease in cardiac fibrosis but also support our previous ones (8) on the decrease of arterial pressure and cardiac hypertrophy in hypertensive rats. Indeed, several studies have shown that relaxin and ANP can reduce arterial blood pressure and cardiac hypertrophy (15, 16, 33, 38). Whether or not there is a link between relaxin and ANP cannot be determined from our present study. Such a link was suggested by the increase in immunoreactive ANP from perfused rat hearts infused with relaxin (39). Yet, the rise in cardiac expression of ANP in response to pressure load was observed even in mice lacking the relaxin peptide (30). Conversely, relaxin administration was shown to exert an anti-fibrotic influence without change in cardiac ANP (10). Thus, if there is a link, it might be indirect and probably not an obligatory condition for the cardiac actions of both peptides. In addition, link between these peptides and oxidative status has been suggested by the inverse correlation found between circulating relaxin or ANP and oxidative stress (reduction of oxidation products but induction of endogenous SOD) in the heart and other organs (40, 41).

In conclusion, the beneficial effects of the specific melon concentrate supplementation observed in SHR-induced cardiac disorders, that is, anti-hypertrophic and anti-fibrotic properties could therefore be explained by the induction of antioxidant defenses as well as restoration of ANP and relaxin pathway in cardiac tissue.
